# Medulloblastoma in a 6 Year Old Mixed Breed Dog: Surgical Debulking and Chemotherapy

**DOI:** 10.3389/fvets.2019.00401

**Published:** 2019-11-14

**Authors:** Rachel Lampe, Miranda D. Vieson, Devon Hague, Dana Connell, Kari Foss, Kim A. Selting

**Affiliations:** ^1^Department of Veterinary Clinical Medicine, University of Illinois at Urbana-Champaign, Urbana, IL, United States; ^2^Veterinary Diagnostic Laboratory, College of Veterinary Medicine, University of Illinois, Urbana, IL, United States

**Keywords:** neuroendocrine, cytoreductive, medulloblastoma, brain tumor, chemotherapy

## Abstract

A medulloblastoma was surgically debulked from a 6 year old American Staffordshire Terrier, who then received a modified lomustine (CCNU), vincristine, procarbazine, and prednisolone (LOPP) protocol. The dog improved significantly and continued to do well until deterioration and euthanasia 5 months following surgery. This is the first known published case report of surgical cytoreductive surgery of a medulloblastoma in a dog with documented response to surgery and chemotherapy. Medulloblastoma is a primitive neuroectodermal tumor that is the most common malignant central nervous system (CNS) tumor in children, though it is less common in adults. This case illustrates the value of considering human literature when creating treatment plans for uncommon brain tumors in veterinary patients. Medulloblastoma should be a differential for cerebellar tumors in young to middle aged dogs, and surgery and chemotherapy should be considered.

## Background

Medulloblastomas are the most common malignant brain tumor in children, making up 40% of pediatric posterior fossa tumors (1). They are rare in adults, making up only 1% of central nervous system (CNS) tumors diagnosed (2). While they are malignant and grow rapidly, only about 7% metastasize outside the CNS (3). Medulloblastomas have only been described in the literature in four dogs, all on postmortem examination (4–7). To the best of our knowledge, there are no reports of ante-mortem diagnosis and treatment of a medulloblastoma in canine patients. This case report describes the neurodiagnostic work up, surgical debulking, and response to chemotherapy in a 6 year old dog.

## Case Presentation

A 6-year-old (28.9 kg) male intact American Staffordshire terrier presented for investigation of a 3 week history of progressive ataxia, falling down stairs, and a left head tilt. Episodes of opisthotonus occurred while the patient was sleeping, and the dog would sometimes appear minimally aware of its surroundings. On presentation the dog was weakly ambulatory and frequently knuckled in the thoracic limbs. Neurological examination identified delayed postural reactions on the left side, absent postural reactions on the right side, and a moderate left head tilt. The remainder of the neurological examination was unremarkable, including spinal reflexes and cranial nerves. The neuroanatomical localization was consistent with a right central (paradoxical) vestibular lesion. A few hours after presentation, the patient rapidly declined. He became laterally recumbent with a decerebrate posture, and his heart rate dropped to 90 bpm, while his blood pressure increased to 170–175 mmHg. Due to the suspected elevated intracranial pressure, a dose of mannitol was administered intravenously (1 g/kg). The patient was premedicated with butophanol (0.2 mg/kg IV) and general anesthesia was induced and maintained with propofol (induction: 4 mg/kg IV) in preparation for magnetic resonance imaging (MRI) of the brain (Siemens Skyra 3T).

**Figure 1:**
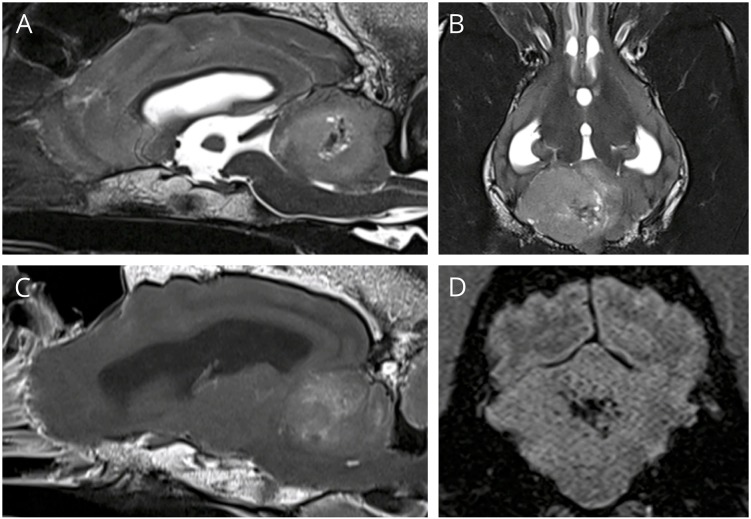
**(A,B)** T2W sagittal and dorsal, showing a large hyperintense space occupying mass in the cerebellum. **(C)** T1 post contrast sagittal, showing mild heterogeneous contrast enhancement, especially at the rim, **(D)** SWI transverse showing signal drop out centrally, consistent with hemorrhage.

MRI revealed a 3.1 × 2.4 × 2.3 cm ill-defined mass within the right cerebellar hemisphere (Figure 1). The mass was heterogeneously and moderately hyperintense on T2 and heterogeneously mildly hyperintense on FLAIR, iso- to hypointense on T1, and was mildly heterogeneously contrast enhancing, especially at the rim. There was a region within the mass, just medial to center, appearing hypointense on all sequences (T1, T2, SWI, and FLAIR) consistent with hemorrhage. There was evidence of rostral transtentorial herniation and obstructive hydrocephalus secondary to the large mass effect. While under anesthesia, dexamethasone was administered intravenously (0.15 mg/kg) to help with perilesional inflammation and anesthetic recovery was uneventful. The following morning, the dog was ambulatory with mild tetraparesis and cerebellar tetra-ataxia with severe intention tremors, specifically noted when the patient would eat. Otherwise, the neurological examination was unchanged. The patient was discharged from the hospital the next day on a tapering course of prednisone (starting at 0.7 mg/kg PO q12).

The owners reported the dog was walking better and had fewer episodes of decreased mentation in the next few days following discharge from the hospital. Four days later, the patient was reevaluated and the neurological examination showed ambulatory cerebellovestibular ataxia with hypermetria of the right side, a moderate head tilt (~20 degrees) and intention tremors. The postural reactions were delayed in the right forelimb, while placing was intact in the other limbs. Computed tomography (GE lightspeed 16) of the chest and abdomen was performed under sedation with butorphanol (0.3 mg/kg IV) and propofol (to effect) and no signs of metastasis were seen.

In preparation for surgery the patient was premedicated with methadone (0.2 mg/kg IV) and mannitol (1 g/kg IV) and induced with propofol (4 mg/kg IV) and lidocaine (2 mg/kg IV). Total intravenous anesthesia was maintained using propofol and fentanyl. He was positioned in sternal recumbency with the neck ventroflexed and the pinna of the ears sutured ventrally to the face with one simple interrupted suture each. The dorsal aspect of the head, neck, and ears were clipped and aseptically prepared for surgery using chlorhexidine scrub. Surgical debulking was performed with a modified suboccipital and right caudal rostrotentorial craniectomy. A midline incision was made rostral to the external occipital protuberance extending caudally to the first cervical vertebrae. The subcutaneous muscle was dissected and reflected off to expose the right temporal bone and occipital bone, using monopolar and bipolar cautery, and periosteal elevators. Minimal hemorrhage was observed during the approach, which was easily controlled with cautery. A drill was used to create one window in the suboccipital region of the skull and a second window in the right caudal temporal bone. The nuchal crest between the two windows was drilled to expose the transverse sinus, which was cauterized using bipolar cautery, bone wax, and gelfoam until adequate occlusion was accomplished. Drilling was then completed to connect the two windows and expose the cerebellum. A purple-pink mass was visualized in the right cerebellar hemisphere, extending past midline and ventrally, obstructing any view of normal right cerebellar tissue. A large amount of friable mass was removed in one large piece with careful blunt dissection. The margins laterally in the cerebellum appeared normal, however, the mass appeared to extend ventrally into the brainstem. In order to avoid traumatizing normal tissues, further resection was not performed ventrally.

**Figure 2:**
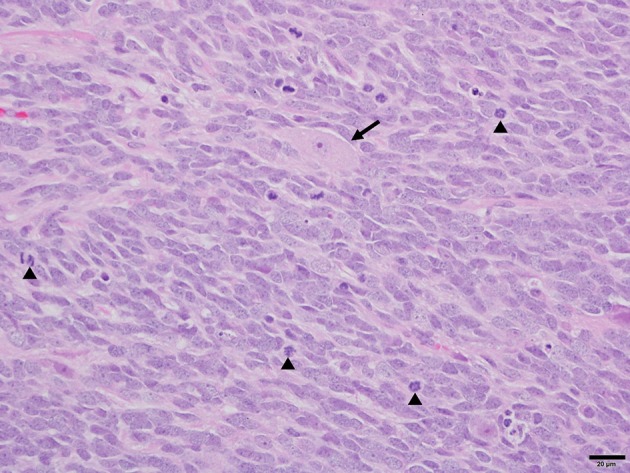
Photomicrograph of medulloblastoma in a dog. Sheets of neoplastic polygonal to elongated cells with large basophilic nuclei and limited pale eosinophilic cytoplasm create the characteristic “small blue tumor” appearance. Further features include a brisk mitotic rate (arrowheads) and rare entrapped cerebellar Purkinje neurons (arrow). H&E. 40× magnification. Bar = 20 um.

Histopathology of the mass revealed a cell-dense mass composed of neoplastic polygonal to elongated cells arranged in dense sheets (Figure 2). The cells had variably indistinct cell borders with a scant amount of pale eosinophilic cytoplasm and a large round to ovoid nucleus. There was moderate anisocytosis and anisokaryosis and a high mitotic rate with 58 mitotic figures in ten 400x fields. Immunohistochemistry (IHC) for glial fibrillary acid protein (GFAP) and synaptophysin was diffusely immunonegative amongst the neoplastic cell population. Rare synaptophysin-positive cells were noted with the mass, which may represent residual entrapped purkinje neurons within the cerebellum implying extensive invasion of the neoplasm into the cerebellar folia. Based on the histomorphology and location, a medulloblastoma was considered the most likely diagnosis.

In the days after surgery, the dog significantly improved. The intention tremors decreased in severity, the head tilt improved, and the patient became strongly ambulatory with no support needed. Ten days after surgery, the dog began falling to the right and became non-ambulatory. Bloodwork was unremarkable, including a normal hematocrit. The decline in neurologic status was suspected to be secondary to the remaining tumor, however repeat imaging to rule out other causes was declined. Based upon objective responses in humans with medulloblastoma to lomustine/CCNU, vincristine, procarbazine, and prednisone alone and in various combinations, the dog was administered a dose of oral CCNU (56 mg/m^2^) (8–10) and prednisone was continued. The patient again improved, and was ambulatory 1 week later. Two weeks after CCNU, the dog was administered vincristine (0.54 mg/m^2^ IV) and started on procarbazine (53 mg/m^2^ PO Q24 for 14 days). This LOPP protocol was chosen because it is a well-established combination protocol in dogs with lymphoma, so much is known about tolerability and scheduling, and because the drugs included in this combination have all shown activity in treating medulloblastoma in people. Other drugs sometimes used in the treatment of people with this cancer, such as cisplatin, might have caused unacceptable toxicity in dogs. Protocols used in people are often high-dose and require stem cell or bone marrow rescue, which is not readily available in veterinary medicine (11).

Six weeks after surgery the patient continued to do well. However, the surgical site dehisced at the skin and dorsal layer of muscle. The dog was sedated and the site cleaned and a culture was obtained. A drain was placed and the site was closed again in 3 layers, with suture and staples. The culture grew *Staphylococcus pseudointermedius* which was sensitive to several antimicrobials. A course of cephalexin (25 mg/kg PO Q12) was administered for 2 weeks. Four weeks later, the patient presented for staple removal. However, the site began to dehisce again after only 1 week, so the staples where replaced.

The dehiscence was suspected to be due to ongoing chemotherapy and prednisone causing delayed healing of the surgical incision. The LOPP protocol was discontinued, the prednisone decreased (0.6 mg/kg PO Q24), and a single agent protocol of CCNU was continued monthly. The patient continued to do very well until 5 1/2 months post-surgery when neurological deterioration occurred and the dog became non-ambulatory. A dose of vincristine (0.68 mg/m^2^ IV) was administered and the dose of prednisone was increased. However, the dog continued to deteriorate and was euthanized a week later. A post mortem examination was not performed.

## Discussion

Medulloblastomas are a common malignant brain tumor in children, however they are rarely reported in the veterinary literature. One review of 435 cases of canine brain tumors diagnosed post-mortem over 25 years described only 6 primitive neuroectodermal tumors and none of these were medulloblastomas (12). Additionally, another study spanning 25 years of brain tumors in immature dogs did not include any medulloblastomas (13). Medulloblastomas have only been described in the literature on postmortem examination in four dogs. In children, medulloblastomas tend to be located medially in the cerebellum, while in adults they often arise laterally from the cerebellar hemispheres (14). In this case report, the location of the mass arising from the lateral hemisphere was similar to the adult form of medulloblastoma described in humans, as was the case in three of the 4 case reports of dog medulloblastomas (4, 5, 7). The third case report was in a 7 year old dog, and the mass arose from the cerebellar vermis (6).

MRI features of medulloblastomas are variable in both adults and children. In humans the masses often have well-defined margins with heterogeneous contrast enhancement, and many have evidence of cysts, peritumoral edema, and hemorrhage (15). These masses often extend to the surface of the brain, and thus may appear extra-axial, like the more common meningioma. The MRI for the dog reported here had many of these characteristics, with heterogeneous mild contrast enhancement, and evidence of hemorrhage and peritumoral edema, although in this dog the margins where poorly defined. The mass appeared intra-axial, however its margins extended to the surface of the cerebellum, which is common in adult medulloblastomas. Determining intra vs. extra-axial location can be important for determining appropriate differential diagnosis and thus prognosis and treatment options. Intra-axial masses are more difficult to debulk surgically, and tend to have a shorter median survival time (16).

Histopathology of this tumor revealed polygonal to elongated cells, with moderate anisocytosis and anisokaryosis, and had negative IHC staining for GFAP and synaptophysin in the neoplastic cell population. One published abstract looking at IHC of 7 primitive canine neuroectodermal tumors found 2/7 stained positive for GFAP, with just one positive for both GFAP and synaptophysin (17). Another series of canine neuroectodermal brain tumors included two canine medulloblastomas which where both negative for GFAP (18). Primitive neuroepithelial cells do not express either synaptophysin or GFAP. The cells that differentiate along the astrocyte line will begin to express GFAP and synaptophysin, while cells that differentiate into neurons will remain negative for GFAP (19). Thus, these differences in IHC staining likely reflect levels of differentiation amongst the tumors, and implies the tumor in this case may be poorly differentiated.

In humans, the standard of care for treatment of medulloblastoma is a combination of surgery, radiation therapy (RT), and often chemotherapy (11). Survival of pediatric patients with medulloblastoma is positively correlated with extent of surgical resection and radiation, and negatively correlated with metastasis (20, 21). Pediatric patients have an overall 5 year survival rate of 44.5–86% (21–23), while adults have an overall 5 year survival rate of 73–80% (24). The addition of RT to the standard treatment protocol in the 1990s was associated with a significant increase in the overall cure rate (21, 22). Adjuvant chemotherapy is routinely used in pediatric medulloblastomas, and more recent papers have shown a likely benefit in adult tumors as well (9). In one study using adjuvant chemotherapy in 15 adults, the survival rates at 15 years was 100% (10). Multiple chemotherapy protocols have been utilized in humans, most commonly including a combination of cisplatin, vincristine, and either lomustine, cyclophosphamide, or etoposide (8, 9, 22).

Radiation therapy was recommended for the dog in this case report after surgery, as it has been shown to improve survival in humans with medulloblastomas (14, 22). However, it was declined due to lack of local availability. A systematic review of brain tumor treatment in dogs has not shown a difference in survival between surgery and radiation therapy when both are an option (16). The dog in this case report initially improved following surgery, then started showing deterioration again 2 weeks later. At this point, a modified LOPP protocol was initiated and the dog showed significant improvement that lasted about 4 months. We hypothesize that the improvement was due to the initiation of chemotherapy, however we cannot rule out that the deterioration and improvement was secondary to intracranial hemorrhage that resolved, as repeat imaging was declined. The average survival for dogs with brain tumors after surgery or radiation therapy is variable, with a median survival time of 300 days (16). However, both intra-axial and infra-tentorial location, as in this dog, are associated with a shorter survival time (16, 25).

This is the first published case report of a dog histologically diagnosed with a medulloblastoma for which surgery and chemotherapy were administered and responses documented. Medulloblastoma should be a differential for both intra and extra-axial cerebellar masses in any age dog. If a medulloblastoma is suspected, chemotherapy should be considered as an adjuvant treatment option, along with surgery and or radiation therapy.

## Data Availability Statement

All datasets for this study are included in the article/supplementary material.

## Ethics Statement

Ethical review and approval was not required for the animal study because not required for retrospective case report. Written informed consent was obtained from the owners for the participation of their animals in this study.

## Author Contributions

RL helped manage patient from diagnosis through euthanasia, conceived of the case report, drafted the article, critically revised the article, and gave final approval of version to be published. MV interpreted the histopathology, critically revised the article, and gave final approval of the version to be published. DH performed the surgery, helped manage patient from diagnosis through euthanasia, critically revised the article, and gave final approval of the version to be published. DC and KS helped manage chemotherapy for patient, critically revised the article, and gave final approval of the version to be published. KF helped with patient management, critically revised the article, and gave final approval of the version to be published. KS provided chemotherapy recommendations, critically revised the article, and gave final approval of the version to be published.

### Conflict of Interest

The authors declare that the research was conducted in the absence of any commercial or financial relationships that could be construed as a potential conflict of interest.

## References

[1] Ostrom QT Gittleman H Xu J Kromer C Wolinsky Y Kruchko C. CBTRUS statistical report: primary brain and other central nervous system tumors diagnosed in the United States in 2009–2013. Neuro Oncol. (2016) 18 (suppl_5):v1–v75. 10.1093/neuonc/now207 28475809 PMC8483569

[2] Koci TM Chiang F Mehringer CM Yuh WT Mayr NA Itabashi H. Adult cerebellar medulloblastoma: imaging features with emphasis on MR findings. AJNR Am J Neuroradiol. (1993) 14:929–39. 8179647 PMC8333838

[3] Rochkind S Blatt I Sadeh M Goldhammer Y. Extracranial metastases of medulloblastoma in adults: literature review. J Neurol Neurosurg Psychiatry. (1991) 54:80–6. 10.1136/jnnp.54.1.80 2010766 PMC1014307

[4] Fraser McConnell J Platt S Smith KC. Magnetic resonance imaging findings of an intracranial medulloblastoma in a Polish Lowland Sheepdog. Vet Radiol Ultrasound. (2004) 45:17–22. 10.1111/j.1740-8261.2004.04003.x 15005356

[5] Steinberg H Galbreath EJ. Cerebellar medulloblastoma with multiple differentiation in a dog. Vet Pathol. (1998) 35:543–6. 10.1177/030098589803500611 9823598

[6] Patsikas M Jakovljevic S Papadopoulou P Polizopoulou Z Kazakos G Tontis D. Magnetic resonance imaging features of cerebellar vermis medulloblastoma in an adult canine patient. J Biol Regul Homeost Agents. (2014) 28:341–7. 25001666

[7] Mandrioli L Biserni R Panarese S Morini M Gandini G Bettini G. Immunohistochemical profiling and telomerase activity of a canine medulloblastoma. Vet Pathol. (2011) 48:814–6. 10.1177/0300985810390016 21123861

[8] Packer RJ Gajjar A Vezina G Rorke-Adams L Burger PC Robertson PL. Phase III study of craniospinal radiation therapy followed by adjuvant chemotherapy for newly diagnosed average-risk medulloblastoma. J Clin Oncol. (2006) 24:4202–8. 10.1200/JCO.2006.06.4980 16943538

[9] Kann BH Lester-Coll NH Park HS Yeboa DN Kelly JR Baehring JM Adjuvant chemotherapy and overall survival in adult medulloblastoma. Neuro Oncol. (2016) 17:now150–11. 10.1093/neuonc/now150 PMC5464064 27540083

[10] Franceschi E Bartolotti M Paccapelo A Marucci G Agati R Volpin L. Adjuvant chemotherapy in adult medulloblastoma: is it an option for average-risk patients? J Neuro Oncol. (2016) 128:235–40. 10.1007/s11060-016-2097-x 26940908

[11] Thomas A Noël G. Medulloblastoma: optimizing care with a multidisciplinary approach. J Multidiscip Healthc. (2019) 12:335–47. 10.2147/JMDH.S167808 31118657 PMC6498429

[12] Song RB Vite CH Bradley CW Cross JR. Postmortem evaluation of 435 cases of intracranial neoplasia in dogs and relationship of neoplasm with breed, age, and body weight. J Vet Intern Med. (2013) 27:1143–52. 10.1111/jvim.12136 23865437

[13] Keller ET Madewell BR. Locations and types of neoplasms in immature dogs: 69 cases (1964-1989). J Am Vet Med Assoc. (1992) 200:1530–2. 1612996

[14] Brandes AA Palmisano V Monfardini S. Medulloblastoma in adults: clinical characteristics and treatment. Cancer Treat Rev. (1999) 25:3–12. 10.1053/ctrv.1998.0096 10212586

[15] Zhao F. Distinctive localization and MRI features correlate of molecular subgroups in adult medulloblastoma. J Neuro Oncol. (2017)135:353–60. 10.1007/s11060-017-2581-y 28808827

[16] Hu H Barker A Harcourt-Brown T Jeffery N. Systematic review of brain tumor treatment in dogs. J Vet Intern Med. (2015) 29:1456–63. 10.1111/jvim.13617 26375164 PMC4895648

[17] An immunohistochemical study of primitive neuroectodermal tumors (PNETs) and olfactory neuroblastomas in dogs and cats In: Abstracts 47th Annual Meeting of American College of Veterinary Pathologists (2009). p. 1–63.

[18] Vandevelde M Fankhauser R Luginbühl H. Immunocytochemical studies in canine neuroectodermal brain tumors. Acta Neuropathol. (1985) 66:111–6. 10.1007/BF00688685 2409734

[19] Fung KM Trojanowski JQ. Animal models of medulloblastomas and related primitive neuroectodermal tumors. A review. J Neuropathol Exp Neurol. (1995) 54:285–96. 10.1097/00005072-199505000-00001 7745427

[20] Michiels EM Schouten-Van Meeteren AY Doz F Janssens GO van Dalen EC Chemotherapy for children with medulloblastoma. Cochrane Database Syst Rev. (2015) 17:301–42. 10.1002/14651858.CD006678.pub2 PMC10651941 25879092

[21] Weil AG Wang AC Westwick HJ Ibrahim GM Ariani RT Crevier L. Survival in pediatric medulloblastoma: a population-based observational study to improve prognostication. J Neuro Oncol. (2017) 132:99–107. 10.1007/s11060-016-2341-4 27981412

[22] Bleil CB Bizzi JWJ Bedin A de Oliveira FH Antunes ÁCM. Survival and prognostic factors in childhood medulloblastoma: a Brazilian single center experience from 1995 to 2016. Surg Neurol Int. (2019) 10:120–9. 10.25259/SNI-237-2019 31528456 PMC6744760

[23] Packer RJ. Risk-adapted craniospinal radiotherapy followed by high-dose chemotherapy and stem-cell rescue in children with newly diagnosed medulloblastoma. Curr Neurol Neurosci Rep. (2007) 7:130, 132. 10.1007/s11910-007-0007-5 17324362

[24] Brandes AA Franceschi E Tosoni A Blatt V Ermani M. Long-term results of a prospective study on the treatment of medulloblastoma in adults. Cancer. (2007) 110:2035–41. 10.1002/cncr.23003 17823910

[25] Rossmeisl JH Jones JC Zimmerman KL Robertson JL. Survival time following hospital discharge in dogs with palliatively treated primary brain tumors. J Am Vet Med Assoc. (2013) 242:193–8. 10.2460/javma.242.2.193 23276095

